# Metabolic Network Analysis and Metatranscriptomics Reveal Auxotrophies and Nutrient Sources of the Cosmopolitan Freshwater Microbial Lineage acI

**DOI:** 10.1128/mSystems.00091-17

**Published:** 2017-08-29

**Authors:** Joshua J. Hamilton, Sarahi L. Garcia, Brittany S. Brown, Ben O. Oyserman, Francisco Moya-Flores, Stefan Bertilsson, Rex R. Malmstrom, Katrina T. Forest, Katherine D. McMahon

**Affiliations:** a Department of Bacteriology, University of Wisconsin—Madison, Madison, Wisconsin, USA; b Department of Ecology and Genetics, Uppsala University, Uppsala, Sweden; c Department of Civil and Environmental Engineering, University of Wisconsin—Madison, Madison, Wisconsin, USA; d Science for Life Laboratory, Uppsala University, Uppsala, Sweden; e United States Department of Energy Joint Genome Institute, Walnut Creek, California, USA; Gladstone Institutes

**Keywords:** freshwater microbial ecology, metabolism, metagenomics, metatranscriptomics, physiology, systems biology

## Abstract

An explosion in the number of available genome sequences obtained through metagenomics and single-cell genomics has enabled a new view of the diversity of microbial life, yet we know surprisingly little about how microbes interact with each other or their environment. In fact, the majority of microbial species remain uncultivated, while our perception of their ecological niches is based on reconstruction of their metabolic potential. In this work, we demonstrate how the “seed set framework,” which computes the set of compounds that an organism must acquire from its environment (E. Borenstein, M. Kupiec, M. W. Feldman, and E. Ruppin, Proc Natl Acad Sci U S A 105:14482–14487, 2008, https://doi.org/10.1073/pnas.0806162105), enables computational analysis of metabolic reconstructions while providing new insights into a microbe’s metabolic capabilities, such as nutrient use and auxotrophies. We apply this framework to members of the ubiquitous freshwater actinobacterial lineage acI, confirming and extending previous experimental and genomic observations implying that acI bacteria are heterotrophs reliant on peptides and saccharides. We also present the first metatranscriptomic study of the acI lineage, revealing high expression of transport proteins and the light-harvesting protein actinorhodopsin. Putative transport proteins complement predictions of nutrients and essential metabolites while providing additional support of the hypothesis that members of the acI are photoheterotrophs.

**IMPORTANCE** The metabolic activity of uncultivated microorganisms contributes to numerous ecosystem processes, ranging from nutrient cycling in the environment to influencing human health and disease. Advances in sequencing technology have enabled the assembly of genomes for these microorganisms, but our ability to generate reference genomes far outstrips our ability to analyze them. Common approaches to analyzing microbial metabolism require reconstructing the entirety of an organism’s metabolic pathways or performing targeted searches for genes involved in a specific process. This paper presents a third approach, in which draft metabolic reconstructions are used to identify compounds through which an organism may interact with its environment. These compounds can then guide more-intensive metabolic reconstruction efforts and can also provide new hypotheses about the specific contributions that microbes make to ecosystem-scale metabolic processes.

## Abstract (precis)

The metabolic activity of uncultivated microorganisms contributes to numerous ecosystem processes, ranging from nutrient cycling in the environment to influencing human health and disease. Advances in sequencing technology have enabled the assembly of genomes for these microorganisms, but our ability to generate reference genomes far outstrips our ability to analyze them. Common approaches to analyzing microbial metabolism require reconstructing the entirety of an organism’s metabolic pathways or performing targeted searches for genes involved in a specific process. This paper presents a third approach, in which draft metabolic reconstructions are used to identify compounds through which an organism may interact with its environment. These compounds can then guide more-intensive metabolic reconstruction efforts and can also provide new hypotheses about the specific contributions that microbes make to ecosystem-scale metabolic processes.

## INTRODUCTION

Natural microbial communities have central roles in the biosphere, ranging from mediating nutrient cycling to influencing human health and disease (1, 2). However, the majority of microbial species remain uncultivated, a state of affairs that poses a significant challenge to our understanding of their physiology and metabolism. Recent advances in sequencing technology and bioinformatics have enabled assembly and analysis of reference genomes for a wide range of hitherto-uncultured community members from diverse environments (3) that can be used to reconstruct an organism’s metabolism.

Common approaches to metabolic reconstruction involve the comprehensive reconstruction of an organism’s metabolic pathways (4) or a targeted search for genes involved in processes of interest (5). These reconstructions can then be analyzed using manual methods or computational approaches such as flux-balance analysis (FBA) (6). However, FBA-based approaches require a comprehensive understanding of an organism’s growth requirements and biomass composition, information which is often unavailable for uncultivated microorganisms. An alternative approach is to compute an organism’s seed set, representing the set of compounds that the organism cannot synthesize on its own and must exogenously acquire from its environment (e.g., its growth requirements) (7). These compounds may represent both auxotrophies, i.e., the essential metabolites for which biosynthetic routes are missing, and nutrients, i.e., the compounds for which degradation routes but not synthesis routes are present in the genome. The seed set framework offers potential advantages over other reconstruction-based approaches, as identification of seed compounds facilitates a focused analysis by identifying those compounds through which an organism interacts with its environment.

In the present report, we present a computational pipeline to predict seed compounds using metabolic network reconstructions generated from KBase (8). We apply this pipeline to a collection of 36 metagenome-assembled genomes (MAGs) and single-cell genomes (SAGs) from the abundant and ubiquitous freshwater actinobacterial lineage acI, which is thought to have a central role in nutrient cycling in diverse freshwater systems (9–18). The seed compounds predicted by our analysis are in agreement with previous experimental and genomic observations (19–27), confirming the ability of our method to predict an organism’s auxotrophies and nutrient sources.

In particular, we found that members of the acI lineage are auxotrophic for essential vitamins and amino acids and may consume as nutrients a wide array of N-containing compounds (including ammonium, branched-chain amino acids, polyamines, and di- and oligopeptides) as well as mono-, poly-, and oligosaccharides. To complement these predictions, and to understand which pathways dominate active metabolism of acI in its natural environment, we conducted an *in situ* metatranscriptomic analysis of gene expression in the acI lineage. This analysis revealed that the members of the acI lineage express a diverse array of transporters for auxotrophies, nutrients, and other compounds that may contribute to their observed dominance and widespread distribution in a variety of aquatic systems.

## RESULTS

### Phylogenetic affiliation of acI genomes.

**FIG 1:**
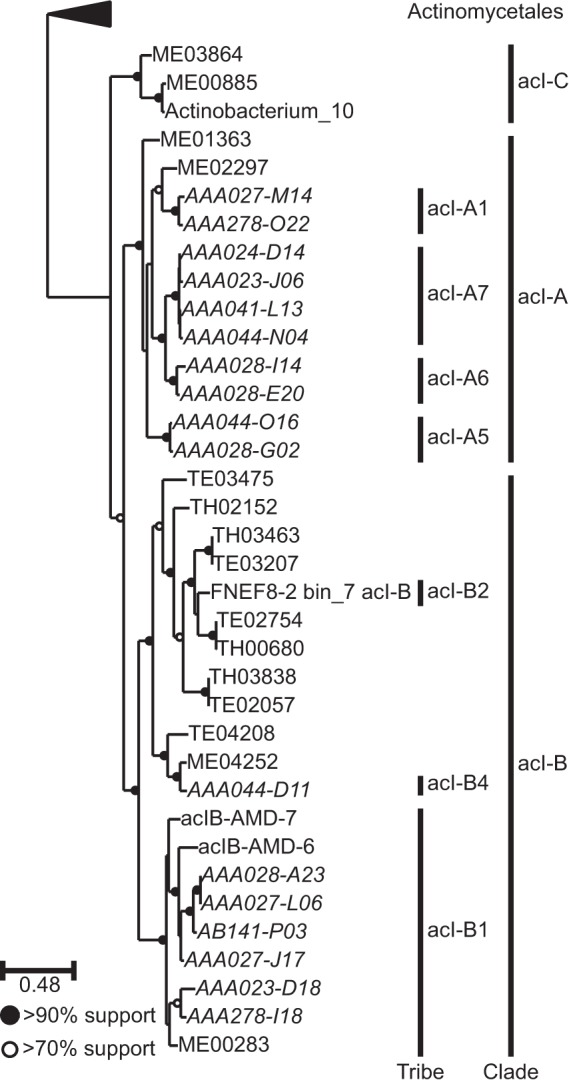
Phylogenetic placement of the genomes used in this study within the acI lineage. The tree was built using RAxML (41) from a concatenated alignment of protein sequences from 37 single-copy marker genes (40). The order *Actinomycetales* forms the outgroup. Vertical black bars indicate groups of genomes belonging to defined tribes/clades within the acI lineage, as determined using 16S rRNA gene sequences (for SAGs and bin FNEF8-2 bin_7 acI-B only) and a defined taxonomy (28). SAGs are indicated with italic text. Figure S1 shows the position of the acI lineage relative to other orders within the class *Actinobacteria*.

We identified 17 SAGs and 19 MAGs from members of the acI lineage (see Table S1 in Data Set S1 in the supplemental material) in a larger set of reference genomes derived from our long-term study sites. A phylogenetic tree of these genomes built using a concatenated alignment of single-copy marker genes is shown in Fig. 1. Previous phylogenetic analyses using 16S rRNA gene sequences showed that the acI lineage can be grouped into 3 distinct monophyletic clades (acI-A, acI-B, and acI-C) and 13 so-called “tribes” (28). In this study, the phylogenetic tree also identified three monophyletic branches, enabling MAGs to be classified to the clade and tribe levels based on the taxonomy of SAGs within each branch (as determined by the 16S rRNA gene sequences that had been either PCR amplified or assembled from the single cell). Note that three MAGs formed a monophyletic group separate from clades acI-A and acI-B; we assume that these genomes belong to clade acI-C as no other acI clades have been identified to date.

10.1128/mSystems.00091-17.8 DATA SET S1  Tables S1 to S19. Download DATA SET S1, XLSX file, 1.3 MB. Copyright © 2017 Hamilton et al. 2017 Hamilton et al. This content is distributed under the terms of the Creative Commons Attribution 4.0 International license.

10.1128/mSystems.00091-17.2 FIG S1  Phylogenetic placement within the acI lineage of the genomes used in this study, relative to other sequenced actinobacterial genomes in the class *Actinobacteria* (50) (see Table S19 in Data Set S1). The tree was built using RAxML (41) from a concatenated alignment of protein sequences from 37 single-copy marker genes (40). The class *Acidimicrobiia* forms the outgroup. Vertical black bars indicate groups of genomes belonging to defined tribes/clades within the acI lineage, as determined using 16S rRNA gene sequences (for SAGs and bin FNEF8-2 bin_7 acI-B only) and a defined taxonomy (28). SAGs are indicated with italic text. Download FIG S1, EPS file, 2.6 MB. Copyright © 2017 Hamilton et al. 2017 Hamilton et al. This content is distributed under the terms of the Creative Commons Attribution 4.0 International license.

### Estimated completeness of tribe- and clade-level composite genomes.

We constructed composite genomes from multiple SAGs and/or MAGs to partially alleviate the limitations presented by incomplete genomes. To do this, we first estimated the completeness of tribe- and clade-level composite genomes using CheckM (29), which uses lineage-specific marker genes organized into collocated sets to obtain a robust estimate of genome completeness. This allowed us to determine the finest level of taxonomic resolution at which we could confidently compute seed compounds, using genome completeness as a proxy for metabolic reaction network completeness (see Fig. S2 in the supplemental material). We deemed genomes to be nearly complete if they contained 95% of the lineage-specific marker genes. With the exception of tribe acI-B1, the tribe-level composite genomes were estimated to be incomplete (Fig. S2A). At the clade level, the genomes of clades acI-A and acI-B are estimated to be nearly complete, while the acI-C composite genome remains incomplete, as it contains only 75% of the 204 marker genes (Fig. S2B). As a result, seed compounds were calculated for composite clade-level genomes, with the understanding that some true seed compounds for the acI-C clade will not be predicted.

10.1128/mSystems.00091-17.3 FIG S2  Mean estimated completeness of tribe-level (clade-level) population genomes as a function of the number of sampled genomes. For each tribe (clade), genomes were randomly sampled (with replacement) from the set of all genomes belonging to that tribe (clade). Completeness was estimated using 204 single-copy marker genes from members of the phylum *Actinobacteria* (29). Error bars represent the 95% confidence interval estimated from 1,000 iterations. Download FIG S2, EPS file, 1.3 MB. Copyright © 2017 Hamilton et al. 2017 Hamilton et al. This content is distributed under the terms of the Creative Commons Attribution 4.0 International license.

### Computation and evaluation of potential seed compounds.

**FIG 2:**
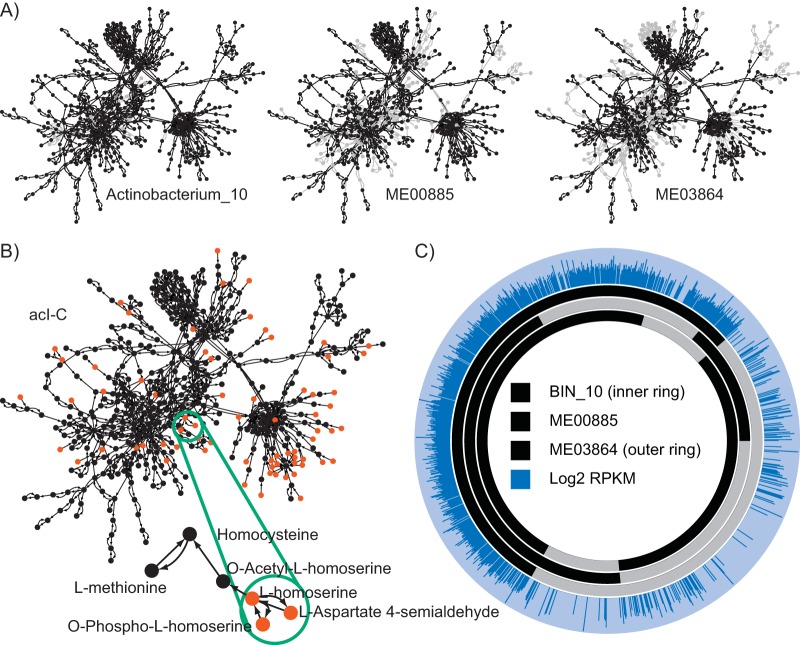
Overview of the seed set framework and metatranscriptomic mapping, using three genomes from the acI-C clade as an example. (A) Metabolic network graphs were created for each genome belonging to clade acI-C. In these graphs, metabolites are represented as nodes (circles) and reactions by arcs (arrows). Gray nodes and edges indicate components of the composite graph missing from that genome graph. Additional information on this step of the workflow is available in Fig. S2. (B) A composite network graph was created for each clade by joining graphs representing all genomes from that clade, and seed compounds (red) were computed for the composite graph. Additional information on this step of the workflow is available in Fig. S3, Fig. S4, and Fig. S5. **(**Inset**)** Three seed compounds which indicate an auxotrophy for l-homoserine, a methionine precursor. (C) Metatranscriptomic reads were mapped to each individual genome using BBMap. Orthologous gene clusters were identified using OrthoMCL (30). For each cluster, unique reads which map to any gene within that cluster were counted using HTSeq (48). The relative levels of gene expression were computed using RPKM (49).

Metabolic network reconstructions for each genome were built using KBase. Composite metabolic network graphs were then constructed for each tribe and clade by merging metabolic network reconstructions of individual genomes. Seed compounds for each clade were then computed from that clade’s composite metabolic network graph using a custom implementation of the seed set framework (Fig. 2). A total of 125 unique seed compounds were identified across the three clades (Table S2 in Data Set S1).

10.1128/mSystems.00091-17.4 FIG S3  Converting an unannotated genome to a metabolic network graph, for a simplified genome containing only glycolysis. (A) Microbial contigs were annotated using KBase, and a metabolic network reconstruction was built from the annotations. The reconstruction provides links between protein-encoding genes in the genome and the enzymatic reactions catalyzed by those proteins. (B) The metabolic network reconstruction represents metabolism as a hypergraph, in which metabolites are represented as nodes and reactions as hyperedges. In this representation, an edge can connect more than two nodes. For example, a single hyperedge (denoted by a heavy black line) connects the metabolites glucose and ATP to glucose-6P, ADP, and P_i_. For clarity, protons are not shown. (C) However, the algorithm used by the seed set framework requires metabolism to be represented as a metabolic network graph, in which an edge can connect only two nodes. In this representation, a reaction is represented by a set of edges connecting all substrates to all products. For example, the heavy hyperedge in panel B is now denoted by six separate edges connecting glucose to ADP, glucose to P_i_, glucose to glucose-6P, ATP to ADP, ATP to P_i_, and ATP to glucose-6P (again denoted by heavy black lines). Of these, only one reaction (glucose to glucose-6P) is biologically meaningful. The dotted line surrounds the currency metabolites. (The images in panels B and C were adapted from reference 51 with permission of the publisher.) (D) The metabolic network graph was then pruned, a process which removed all currency metabolites and any edges in which those metabolites participated. Of the six heavy edges in panel C, only the biologically meaningful one, connecting glucose to glucose-6P (again denoted by a heavy black line), was retained. Download FIG S3, EPS file, 2.8 MB. Copyright © 2017 Hamilton et al. 2017 Hamilton et al. This content is distributed under the terms of the Creative Commons Attribution 4.0 International license.

10.1128/mSystems.00091-17.5 FIG S4  Construction of composite metabolic network graph for clade acI-C. Beginning with metabolic network graphs for genomes actinobacterium_10 and ME00885, nodes and edges unique to ME00885 were identified (in blue). These nodes and edges were added to the actinobacterium_10 graph, giving the composite metabolic network graph for these two genomes (actinobacterium_10 plus ME00885). Then, this graph was compared to the graph for ME03864, and nodes and edges unique to ME03864 were identified (in blue). These nodes and edges were added to the metabolic network graph corresponding to actinobacterium_10 plus ME00885, giving the composite metabolic network graph for clade acI-C. Download FIG S4, TIF file, 0.3 MB. Copyright © 2017 Hamilton et al. 2017 Hamilton et al. This content is distributed under the terms of the Creative Commons Attribution 4.0 International license.

10.1128/mSystems.00091-17.6 FIG S5  Identifying seed compounds in metabolic networks, using the same metabolic network as was used for Fig. S3. (A) To identify seed compounds, the metabolic network graph was first decomposed into its strongly connected components (SCCs), sets of nodes such that each node in the set was reachable from every other node. Here, each set of circled nodes corresponds to a unique SCC. (B) SCC decomposition enabled seed sets to be identified from source components (components with no incoming edges) in the condensation of the original graph. In the condensation of the original graph shown here, each node corresponds to a unique SCC. This network has a single seed set, SCC_1, enclosed in a dotted circle. (C) Seed compounds can be found from the mapping between SCCs and their constituent metabolites. In this example, glucose is the sole seed compound. While this particular result is probably intuitive, real metabolic networks are considerably more complex. Note that the visual representations shown here are intended to illustrate the metabolic network reconstruction process and are not indicative of the data structures used by our pipeline. Download FIG S5, EPS file, 1.7 MB. Copyright © 2017 Hamilton et al. 2017 Hamilton et al. This content is distributed under the terms of the Creative Commons Attribution 4.0 International license.

**FIG 3:**
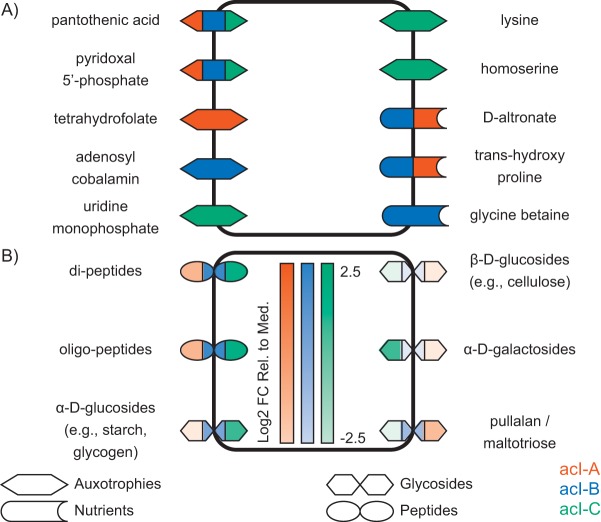
Seed compounds of members of the acI lineage. (A) Auxotrophies and nutrient sources, not including peptides and glycosides. (B) Peptides and glycosides. These compounds represent those inferred from genome annotations rather than the seed compounds. In panel B, the intensity of the color indicates the log_2_ fold change relative to the median (FC Rel. to Med.) of the encoding gene cluster. For compounds acted upon by multiple gene clusters, the percentile of the most highly expressed cluster was chosen.

Because KBase is an automated annotation pipeline, the predicted set of seed compounds is likely to contain inaccuracies (e.g., due to missing or incorrect annotations). As a result, we screened the set of predicted seed compounds to identify those that represented biologically plausible auxotrophies and nutrients and manually curated this subset to obtain a final set of auxotrophies and nutrient sources. Of 125 unique compounds, 31 (24%) were retained in the final set of proposed auxotrophies and nutrients. Tables S3 and S4 in Data Set S1 contain this final set of compounds for clades acI-A, acI-B, and acI-C, and Fig. 3 shows the auxotrophies and nutrients that these compounds represent.

### Making sense of seed compounds via protein clustering and metatranscriptomic mapping.

For seed compounds representing nutrient sources, genes associated with the consumption of these compounds should be expressed. To test this, we collected and sequenced four metatranscriptome samples from Lake Mendota (Dane County, WI, USA). However, because seed compounds were computed from each clade’s composite metabolic network graph, genes associated with the consumption of seed compounds may be present in multiple genomes within the clade. To facilitate the linkage of metatranscriptome measurements to seed compounds, we used OrthoMCL (30) to identify clusters of orthologous groups (COGs) in the set of acI genomes, merged metatranscriptome reads from all four samples, and mapped the reads to COGs within each clade.

Sequencing of cDNA from all four rRNA-depleted metatranscriptome samples yielded approximately 160 million paired-end reads. After merging, filtering, and further *in-silico* rRNA removal, approximately 81 million, or 51%, of the reads remained (Table S5 in Data Set S1). We then used BBMap (https://sourceforge.net/projects/bbmap/) to map metatranscriptome reads to our reference genome collection. After mapping the metatranscriptomes to our acI genomes, we calculated the average coverage of each genome in our reference collection. Within each clade, the most abundant genome was detected with at least 16-fold coverage (Table S6 in Data Set S1).

Finally, we calculated gene expression for each COG on the basis of the number of reads per kilobase per million (RPKM) (Fig. 2). OrthoMCL identified a total of 5,013 protein clusters across the three clades (Table S7 in Data Set S1) with an average confidence of 84% in annotation for COGs containing more than one gene. The COGs were unequally distributed across the three clades, with clade acI-A genomes containing 3,175 COGs (63%), clade acI-B genomes containing 3,459 COGs (69%), and clade acI-C genomes containing 1,365 COGs (27%). Of these, 525 COGs were expressed in clade acI-A, 661 in clade acI-B, and 813 in clade acI-C (Table S8 in Data Set S1). Among the expressed genes, the median log_2_ RPKM values were 31.1 in clade acI-A, 32.0 in clade acI-B, and 69.4 in clade acI-C. Due to differing RPKM values in each clade, we report gene expression values for each clade relative to the median log_2_ RPKM value for that clade.

### Auxotrophies and nutrient sources of the acI lineage.

Seed set analysis yielded seven auxotrophies that could be readily mapped to ecophysiological attributes of the acI lineage (Fig. 3A and Table S3 in Data Set S1). In all three clades, beta-alanine was identified as a seed compound, suggesting an auxotrophy for pantothenic acid (vitamin B5), a precursor to coenzyme A formed from beta-alanine and pantoate (Table S9 in Data Set S1). In bacteria, beta-alanine is typically synthesized via aspartate decarboxylation, and we were unable to identify a candidate gene for this enzyme (aspartate 1-decarboxylase; EC 4.1.1.11) in any acI genome. Pyridoxine 5′-phosphate and 5′-pyridoxamine phosphate (forms of the enzyme cofactor pyridoxal 5′-phosphate [vitamin B6]) were also predicted to be seed compounds, and genes encoding numerous enzymes involved in the biosynthesis of these compounds were not found in the genomes (Table S9 in Data Set S1).

Clades within the acI lineage also exhibited distinct auxotrophies. Clade acI-A was predicted to be auxotrophic for the cofactor tetrahydrofolate (THF [vitamin B9]), and numerous enzymes for its biosynthesis were missing (Table S9 in Data Set S1). This cofactor plays an important role in the metabolism of amino acids and vitamins. In turn, clade acI-B was predicted to be auxotrophic for adenosylcobalamin (vitamin B12), containing only four reactions from its biosynthetic pathway (Table S9 in Data Set S1). Finally, acI-C was predicted to be auxotrophic for the nucleotide UMP (used as a monomer in RNA synthesis) and the amino acids lysine and homoserine. In all cases, multiple enzymes for the biosynthesis of these compounds were not found in the acI-C genomes (Table S9 in Data Set S1).

A number of seed compounds were also predicted to be degraded by members of the acI lineage (Fig. 3B; Table S3 in Data Set S1). Both clade acI-A and clade acI-B were predicted to use d-altronate and *trans*-4-hydroxy proline as nutrients, and acI-B was additionally predicted to use glycine betaine.

Finally, all three clades were predicted to use dipeptides and the sugar maltose as nutrients. Clades acI-A and acI-C were also predicted to consume the polysaccharides stachyose, manninotriose, and cellobiose. In all cases, these compounds were associated with reactions catalyzed by peptidases or glycoside hydrolases (Tables S10 and S11 in Data Set S1), which may be capable of acting on compounds beyond the predicted seed compounds. Thus, we used these annotations to define nutrient sources, rather than using the predicted seed compounds themselves. Among these nutrient sources were di- and polypeptides, predicted to be released from both cytosolic and membrane-bound aminopeptidases. As discussed below, we identified a number of transport proteins capable of transporting these released residues. In Lake Mendota, clades acI-B and acI-C expressed two aminopeptidases, one of which was expressed at nearly 175% of the median gene expression levels (Table S10 in Data Set S1). Clade acI-A expressed a third aminopeptidase at a lower level (40%, the median gene expression level) (Table S10 in Data Set S1).

All three clades were predicted to encode an alpha-glucosidase, which in Lake Mendota was expressed only in clades acI-B and acI-C, at nearly 60% of the median gene expression level (Table S11 in Data Set S1). All three clades also encode a beta-glucosidase, but it was not expressed in our samples. Furthermore, all three clades encode an alpha-galactosidase and multiple maltodextrin glucosidases (which free maltose from maltotriose), but these were expressed only in clades acI-A and acI-C. The alpha-galactosidase had a log_2_ RPKM expression value of 1.5 times the median in clade acI-C, while the maltodextrin glucosidases were expressed at approximately 30% of the median (Table S11 in Data Set S1) in both clade acI-A and clade acI-C.

### Compounds transported by the acI lineage.

Microbes may be capable of transporting compounds that are not strictly required for growth, and comparing such compounds to predicted seed compounds can provide additional information about an organism’s ecology. Thus, we used the metabolic network reconstructions for the acI genomes to systematically characterize the transport capabilities of the members of the acI lineage.

**FIG 4:**
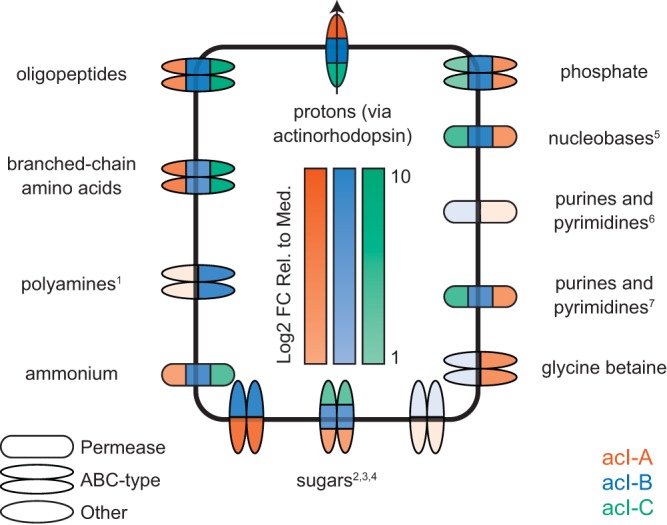
Transporters that are actively expressed by members of the acI lineage, as inferred from consensus annotations of genes associated with transport reactions present in metabolic network reconstructions. The intensity of the color indicates the log_2_ fold change relative to the median value determined for the encoding gene cluster. For multisubunit transporters, the RPKM of the substrate-binding subunit was chosen (see Table S13 in Data Set S1). For some transporters, consensus annotations have been replaced with broad metabolite classes. Such metabolite classes are indicated with superscripts, and the original annotations are as follows: 1, spermidine and putrescine; 2, maltose; 3, xylose; 4, ribose; 5, uracil; 6, cytosine/purine/uracil/thiamine/allantoin; 7, xanthine/uracil/thiamine/ascorbate.

All acI clades encode and were found to express a diverse array of transporters (Fig. 4; Data Set S1; Text S1). Consistent with the presence of peptidases, all clades contained numerous genes for the transport of peptides and amino acids, including putative oligopeptide and branched-chain amino acid transporters, as well as putative transporters for the polyamines spermidine and putrescine. All clades also contained a putative transporter for ammonium. The ammonium, branched-chain amino acid, and oligopeptide transporters had expression values above the median, with expression values for the substrate-binding protein (of the ATP-binding cassette [ABC] transporters) ranging from 1.7 to 411 times the median (Table S13 in Data Set S1). In contrast, while all clades expressed some genes from the polyamine transporters, only clade acI-B expressed the binding protein, at a level approximately 27.8 times the median (Table S13 in Data Set S1). Finally, clades acI-A and acI-B also contain a putative transporter for glycine betaine, which was expressed only in clade acI-A, at approximately 9.6 times the median (Table S13 in Data Set S1). However, we cannot rule out the possibilities that the expression of these transporters changes with space and time and that all three clades may express these enzymes under different conditions.

10.1128/mSystems.00091-17.1 TEXT S1  Supplemental Methods, Results, and Discussion. Download TEXT S1, DOCX file, 0.02 MB. Copyright © 2017 Hamilton et al. 2017 Hamilton et al. This content is distributed under the terms of the Creative Commons Attribution 4.0 International license.

All clades also expressed transporters consistent with the presence of glycoside hydrolases, including transporters annotated as putative maltose, xylose, and ribose ABC-type transporters, which may indicate that acI bacteria are capable of transporting sugars, including both disaccharides (maltose) and monosaccharides (xylose and ribose). Of these, the putative maltose transporter was most highly expressed (but only in clades acI-A and acI-B), with expression values for the substrate-binding protein in a range in excess of 40 times the median (Table S13 in Data Set S1).

Representatives from the acI lineage were also found to encode and express a number of transporters that do not have corresponding seed compounds, including potential nucleobase transporters and purine/pyrimidine transporters (annotated as a uracil and a xanthine/uracil/thiamine/ascorbate family permease, respectively). Both of these are expressed in all three clades, with expression values ranging from 4.7 to 46 times the median (Table S13 in Data Set S1). Clades acI-A and acI-B also contained a second potential purine/pyrimidine transporter (annotated as a cytosine/purine/uracil/thiamine/allantoin family permease), which was expressed only in clade acI-B (Table S13 in Data Set S1). These transporters may be responsible for the uptake of the seed compounds UMP (a pyrimidine derivative) and vitamin B1 (also known as thiamine). In addition, clade acI-A contained but did not express a putative transporter for cobalamin (vitamin B12), and both clade acI-A and clade acI-B contained but did not express transporters for thiamine (vitamin B1) and biotin (vitamin B7) (Table S13 in Data Set S1).

Finally, all three clades expressed actinorhodopsin, a light-sensitive protein that is expected to function as a proton efflux pump (31). In all clades, actinorhodopsin was among the top 10 most highly expressed genes (Table S7 in Data Set S1), with expression values in excess of 84 times the median in all three clades (Table S7 in Data Set S1). Given that many of the transport proteins are ABC transporters, we speculate that actinorhodopsin may facilitate maintenance of the proton gradient necessary for ATP synthesis. Coupled with high expression levels of diverse transporters, this result strongly suggests that acI functions as a photoheterotroph. However, it remains to be seen if this behavior is a general feature of acI physiology or if it is restricted to the specific conditions of the lake and our sampling period.

## DISCUSSION

This study used high-throughput metabolic network reconstruction and the seed set framework to predict auxotrophies and nutrient sources of uncultivated microorganisms from incomplete genome sequences. The computational approach easily scales to hundreds of metabolic reconstructions and enables a targeted analysis by identifying those compounds through which an organism interacts with its environment. However, predicted seed compounds are sensitive to the metabolic network structure, and analyzing the results requires significant manual curation of the metabolic reconstruction and accurate interpretation of the underlying gene annotations. As a consequence, the seed set framework is not as high throughput as was initially envisioned but is nevertheless suitable for analysis of microorganisms with high-quality metabolic network reconstructions.

Our predictions of the substrate use capabilities of the acI lineage are largely congruent with previous genome-centered studies based on smaller but manually curated genome collections (22, 25, 27), indicating that the use of automatic metabolic network reconstructions yields predictions similar to metabolic reconstruction efforts that are based on more extensively manual methods, while being both more high throughput and more focused on an organism’s substrate utilization capabilities. In particular, this study predicted that the consumption of N-rich compounds is a universal feature of the acI lineage, with all three clades predicted to consume ammonium, branched-chain amino acids, polyamines, and di- and oligopeptides. These findings agree with the results of microautoradiography-fluorescent *in situ* hybridization (MAR-FISH) studies that confirm the ability of acI bacteria to consume a variety of amino acids (20, 23). Furthermore, the presence of alpha- and beta-glucosidases is consistent with observations that acI bacteria consume glucose (19, 23), even though no obvious glucose transport system was found in the genomes. Because transport proteins are often capable of acting on multiple substrates, one of the putative sugar transporters may be responsible for glucose uptake activity.

However, our approach failed to recapitulate other genomic and experimental observations, including the uptake of N-acetylglucosamine (NAG) (32–34), the deoxynucleoside thymidine (23, 35), and acetate (19) as well as the potential to hydrolyze the cyanobacterial peptide cyanophycin via the enzyme cyanophycinase (22, 25). Inspection of these discrepancies reveals some important limitations of the seed set framework and automatic metabolic reconstructions. First, the seed set framework identifies only the compounds that the metabolic network must obtain from its environment and fails to identify compounds that the organism can acquire from its environment but can also itself synthesize. Thymidine and acetate fall into this category. Second, automatic metabolic network reconstructions may not fully capture an organism’s metabolic network (e.g., due to missing or incorrect genome annotations). Manual inspection of the product of the previously identified cyanophycinase gene revealed that KBase annotated this putative enzyme as a hypothetical protein. As biochemical characterization of hypothetical proteins and automatic gene and protein annotation are active areas of research, we anticipate that advances in these fields will continue to improve the accuracy of automatic metabolic network reconstructions.

This study also suggested that auxotrophies for some vitamins may be universal features of the acI lineage, as we predicted all clades to be auxotrophic for pantothenic acid and pyridoxal 5′-phosphate (vitamins B5 and B6). We also predict new auxotrophies within the acI lineage, including THF (clade acI-A), adenosylcobalamin (vitamin B12; clade acI-B), and lysine, homoserine, and UMP (clade acI-C). However, with the exception of adenosylcobalamin, we did not identify transporters for any of these compounds. This negative result may reflect our limited knowledge of transport proteins (36); transporters for these compounds may yet be present in the genomes, or one or more of the predicted transporters may act on these compounds. Furthermore, because the acI-C composite genome was estimated to be around 75% complete, we cannot rule out the possibility that the missing genes might be found in this clade when additional genomes are recovered. Nonetheless, these results provide additional support for the hypothesis that distributed metabolic pathways and metabolic complementarity may be common features of freshwater bacterial communities (37, 38).

Combined, these results suggest that the members of the acI lineage are photoheterotrophs and that their survival depends on the availability of a diverse array of N-rich compounds, saccharides, and light. The acI lineage does not appear to be metabolically self-sufficient and may participate in the turnover of high-molecular-weight dissolved organic compounds, such as starch, glycogen, and cellulose. Metatranscriptomic analysis showed that transport proteins were among those most highly expressed in the acI genomes, and expression of multiple putative amino acid transporters may facilitate uptake of these labile compounds. We also observed differences in the relative levels of expression of these transporters, which may point to clade-specific differences in affinities for these substrates. Finally, the actinorhodopsin protein was highly expressed and may facilitate synthesis of the ATP needed to drive acI’s many ABC-type transporters.

Finally, the fragmented and incomplete nature of SAGs and MAGs required us to construct composite genomes for individual acI clades by leveraging multiple genomes from closely related populations. Such an approach limits the resolution of predictions, as we cannot make predictions at the level of tribes, smaller populations, or individual cells. Thus, metabolic diversification at these taxonomic levels would be missed. Constructing composite genomes may also overestimate the metabolic capabilities of a clade or group; for example, if a complete pathway is present in a clade but is distributed among different tribes, the clade would be able to carry out the activity of the entire pathway *in situ* only if all tribes were present in close enough proximity to exchange pathway intermediates. Nonetheless, the seed set approach provides a framework that can be used to generate new hypotheses about the substrates used by members of a defined phylogenetic group, provided that multiple closely related genomes are available. As metagenomic assembly and binning techniques and single-cell sequencing methods improve and complete genomes become available, we anticipate our approach being applied to individual microbial genomes.

## MATERIALS AND METHODS

### A freshwater reference genome collection.

This study relied on an extensive collection of freshwater bacterial genomes containing MAGs obtained from two metagenomic time series from two Wisconsin lakes (27, 39) as well as SAGs from three lakes in the United States (21). Additional information about this genome collection can be found in Text S1.

### Metatranscriptome sampling and sequencing.

This study used four metatranscriptomes obtained as part of a larger study of gene expression in freshwater microbial communities. Additional information about these samples can be found in the Text S1. All protocols and scripts for sample collection, RNA extraction, rRNA depletion, sequencing, and bioinformatic analysis can be found on Github (https://github.com/McMahonLab/OMD-TOIL [DOI: 10.5281/zenodo.839851]). Metadata for the four samples used in this study can be found in Table S6 in Data Set S1 in the supplemental material, and the raw RNA sequences can be found on the Sequence Read Archive (SRA) of the National Center for Biotechnology Information (see below).

### Identification of acI SAGs and actinobacterial MAGs.

The members of the acI were previously phylogenetically divided into 3 clades (acI-A, acI-B, and acI-C) and 13 tribes on the basis of their 16S rRNA gene sequences (28). The acI SAGs were identified within a previously published genome collection (21) and classified to the tribe level using partial 16S rRNA genes and a reference taxonomy for freshwater bacteria, as described in Text S1. Actinobacterial MAGs were identified within two metagenomic time series (27, 39) using taxonomic assignments from a subset of conserved marker genes, as described in Text S1. Phylogenetic analysis of acI SAGs and actinobacterial MAGs was performed using a concatenated alignment of single-copy marker genes obtained via Phylosift (40). Maximum likelihood trees were generated using RAxML (41), the automatic protein model assignment option (PROTGAMMAAUTO), and 100 bootstraps.

### Genome annotation, metabolic network reconstruction, and computation and evaluation of seed compounds.

In the seed set framework, an organism’s metabolism is represented via a metabolic network graph, in which nodes denote compounds and edges denote enzymatically encoded biochemical reactions linking substrates and products (42). Allowable biochemical transformations can be identified by drawing paths along the network, in which a sequence of edges connects a sequence of distinct vertices. In our implementation of the seed set framework, metabolic network graphs were generated as follows.

Genome annotations were performed and metabolic network reconstructions were built using KBase. Contigs for each genome were uploaded to KBase and annotated using the “Annotate Microbial Contigs” method with default options, which uses components of the RAST toolkit for genome annotation (43, 44). Metabolic network reconstructions were obtained using the “Build Metabolic Model” app with default parameters, which relies on the Model SEED framework (45) to build a draft metabolic model. To ensure that the reconstructions contained only reactions with genomic evidence, no gap filling was performed. These reconstructions were then pruned of currency metabolites (compounds used to carry electrons and functional groups) and highly connected compounds and converted to metabolic network graphs (see Fig. S3 and Text S1 in the supplemental material). Many of the individual acI genomes are incomplete. Therefore, to increase the accuracy of seed identification by means of the use of a more complete metabolic network, composite metabolic network graphs were constructed for each tribe and clade (Fig. S4; Text S1).

Formally, the seed set of the network is defined as the minimal set of compounds that cannot be synthesized from other compounds in the network and whose presence enables the synthesis of all other compounds in the network (7). Seed compounds for each composite metabolic network graph were calculated using a new Python implementation of the seed set framework (7) (Fig. S5 and Text S1). Because seed compounds are computed from a metabolic network, it is important to manually evaluate all predicted seed compounds to identify those that may be biologically meaningful and that do not arise from errors in the metabolic network reconstruction. Compounds involved in fatty acid and phospholipid biosynthesis pathways were removed during curation, as these pathways are often organism specific and unlikely to be properly annotated by automatic metabolic reconstruction pipelines. Seed compounds related to currency metabolites were also removed, as data corresponding to reactions for the synthesis of these compounds may have been removed during network pruning.

Text S1 contains a series of brief vignettes explaining why selected compounds were discarded based on the aforementioned considerations and provides examples of additional curation efforts applied to biologically plausible compounds. For a plausible auxotrophy, we screened the genomes for the canonical biosynthetic pathway(s) for that compound and retained those compounds for which the biosynthetic pathway was incomplete. For identification of a plausible nutrient source, we screened the genomes for the canonical degradation pathway(s) for that compound and retained those compounds for which the degradation pathway was complete.

All computational steps were implemented using Python scripts, freely available as part of the reverseEcology Python package developed for this project (https://pypi.python.org/pypi/reverseEcology/ [DOI: 10.5281/zenodo.839856]).

### Identification of transported compounds.

For each genome, we identified all transport reactions present in its metabolic network reconstruction. Gene-protein-reaction associations (GPRs) for these reactions were manually curated to remove unannotated proteins, to group genes into operons (if applicable), and to identify missing subunits for multisubunit transporters. These genes were then mapped to their corresponding COGs and grouped accordingly. Finally, the most common annotation for each COG was used to identify likely substrates for each of these groups. Only transporters with >50% confidence in the substrate-binding subunit were retained. Because identification and annotation of transport proteins are active areas of research (36), substrates for each transporter are described as putative and acting on molecular classes (e.g., saccharide, amino acid) instead of on specific compounds, in order to better reflect the promiscuity of transport proteins and the ambiguity of their annotation.

### Protein clustering, metatranscriptomic mapping, and clade-level gene expression.

OrthoMCL (30) was used to identify clusters of orthologous groups (COGs) in the set of acI genomes. Both OrthoMCL and BLAST were run using default options (46). Annotations were assigned to protein clusters by choosing the most common annotation among all genes assigned to the respective cluster and a confidence score assigned to each COG (representing the fraction of genes having the most common annotation). Trimmed and merged metatranscriptomic reads from each of the four biological samples were then pooled and mapped to a single reference fasta file containing all acI genomes using BBMap with the *ambig=random* and *minid=0.95* options. The 95% identity cutoff was chosen as this represents a well-established criterion for identifying microbial species using average nucleotide identity (ANI) (47), while combining the *ambig* option with competitive mapping using pooled acI genomes as the reference ensured that the reads mapped to only a single genome. These results were then used to compute the expression of each COG in each clade.

Next, HTSeq-Count (48) was run using the *intersection_strict* option to count the total number of reads that map to each gene in our acI genome collection. After mapping, the list of counts was filtered to remove those genes that did not recruit at least 10 reads. Using the COGs identified by OrthoMCL, the genes that correspond to each COG were then identified.

Within each clade, gene expression was computed for each COG on the basis of the number of reads per kilobase per million (RPKM) (49), while also accounting for different gene lengths within a COG and numbers of mapped reads for each genome within a clade. That is, the RPKM value for a single COG represents the sum of RPKM values for each gene within that COG, normalized to the appropriate gene length and total number of mapped reads. RPKM counts were then normalized to the median level of gene expression within that clade. Finally, the expression data (mapping of transcript reads to genes) were visualized to ensure that the RPKM calculations were based on continuous transcription of each gene.

### Accession number(s).

The raw RNA sequences can be found in the Sequence Read Archive (SRA) of the National Center for Biotechnology Information under BioProject accession no. PRJNA362825.

### Data availability.

All genomic and metatranscriptomic sequences are available through IMG and NCBI, respectively. A reproducible version of the manuscript is available at https://github.com/joshamilton/Hamilton_acI_2017 (DOI: 10.5281/zenodo.839858).

10.1128/mSystems.00091-17.7 FIG S6  Complete composite metabolic network graph for clade acI-C, showing disconnected components (gray) and the single largest component (green and black). Disconnected components were dropped prior to computing the network’s seed sets because these groups of nodes are not connected to the bulk of the network. Within the single largest component, the giant strong component contains a substantial fraction of the compounds (green nodes), giving rise to a bow tie structure in the metabolic network graph. Download FIG S6, EPS file, 2.3 MB. Copyright © 2017 Hamilton et al. 2017 Hamilton et al. This content is distributed under the terms of the Creative Commons Attribution 4.0 International license.
