# Supplementary figures and images for: Metabolic Network Analysis and Metatranscriptomics Reveal Auxotrophies and Nutrient Sources of the Cosmopolitan Freshwater Microbial Lineage acI
Source: mSystems. 2017 Aug 29;2(4):e00091-17. doi: 10.1128/mSystems.00091-17 (PMC5574706; doi:10.1128/mSystems.00091-17)

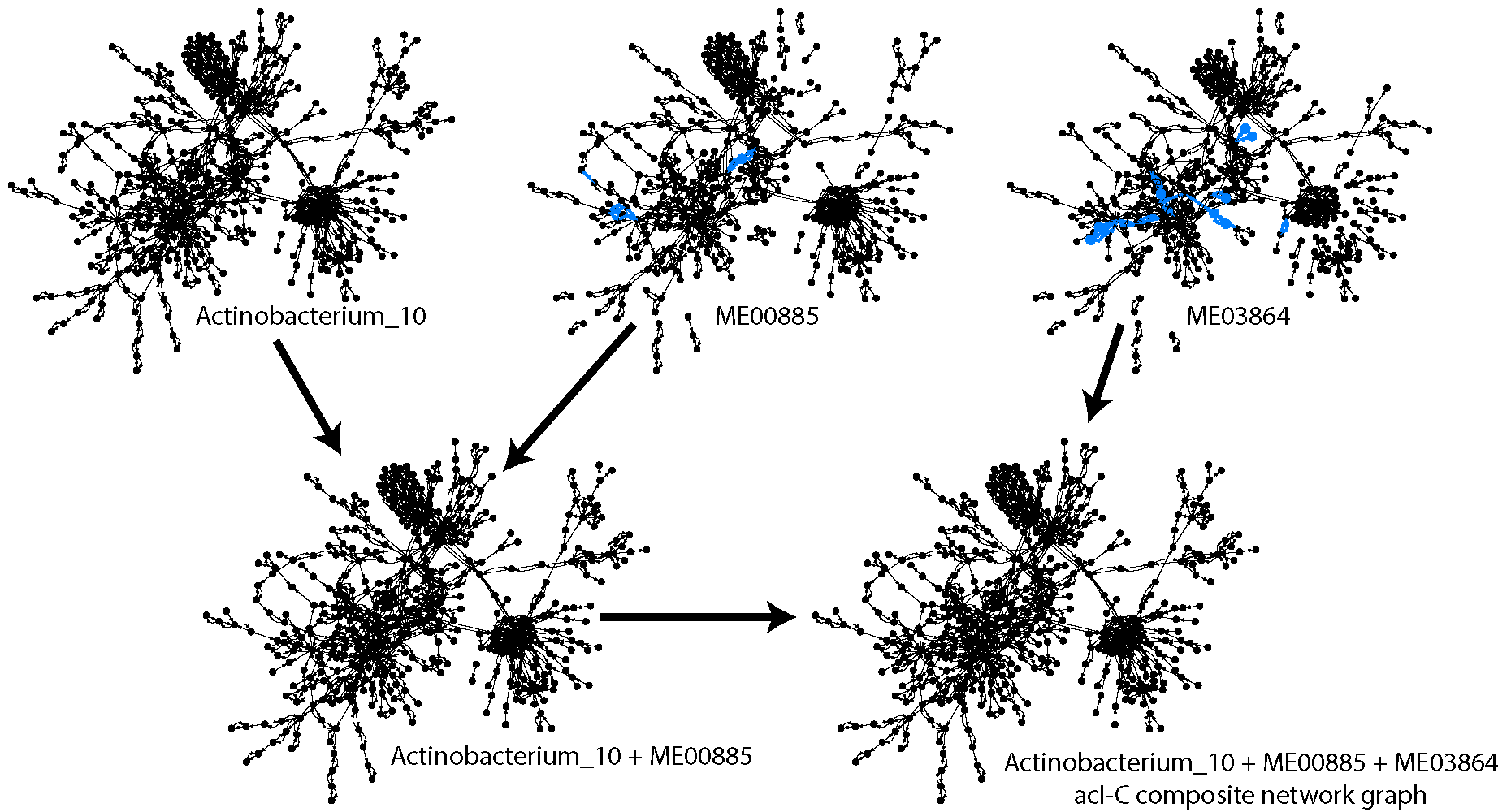

Supplement: FIG S4 [file sys005172131sf6.tif]
