# Supplementary material for: Metabolic Network Analysis and Metatranscriptomics Reveal Auxotrophies and Nutrient Sources of the Cosmopolitan Freshwater Microbial Lineage acI
Source: mSystems. 2017 Aug 29;2(4):e00091-17. doi: 10.1128/mSystems.00091-17 (PMC5574706; doi:10.1128/mSystems.00091-17)
Supplement: TEXT S1 [file sys005172131s1.docx]

Supplemental Material for ‘Metabolic Network Analysis and Metatranscriptomics Reveals Auxotrophies and Nutrient Sources of the Cosmopolitan Freshwater Microbial Lineage acI’

Joshua J. Hamilton^1*^, Sarahi L. Garcia^2, Brittany S. Brown^1, Ben O. Oyserman^3, Francisco Moya-Flores^3, Stefan Bertilsson^2,4, Rex R. Malmstrom^5, Katrina T. Forest^1, Katherine D. McMahon^1,3

^1^ Department of Bacteriology, University of Wisconsin-Madison, Madison, WI, USA; ^2^ Department of Ecology and Genetics, Uppsala University, Uppsala, Sweden; ^3^ Department of Civil and Environmental Engineering, University of Wisconsin-Madison, Madison, WI, USA; ^4^ Science for Life Laboratory, Uppsala University, Uppsala, Sweden; ^5^ United States Department of Energy Joint Genome Institute, Walnut Creek, CA, USA

* Correspondence: Joshua J. Hamilton, jjhamilton2@wisc.edu

# Supplementary Methods

## A Freshwater Reference Genome Collection

This study relies on an extensive collection of freshwater bacterial genomes, containing metagenome-assembled genomes (MAGs) obtained from two metagenomic time-series from two Wisconsin lakes, as well as single-cell genomes (SAGs) from three lakes in the United States. Additional information about this genome collection can be found below.

### Single-Cell Genome Generation, Classification, and Sequencing

Water samples were collected from the top of the water column (depth <1m) from each of three lakes, Lake Mendota (Madison, WI, USA), Sparkling Lake (Vilas County, WI, USA), and Damariscotta Lake (Lincoln County, ME, USA), in 2009. Samples were cryopreserved and sent to the Single Cell Genomics Center at the Bigelow Laboratory for Ocean Sciences for sorting, as previously described (1, 2). Partial 16S rRNA genes amplified previously (1) were phylogenetically classified using a controlled nomenclature for freshwater bacteria (3) by insertion into references trees created in the ARB software package (4).

Actinobacterial SAGs used in this study were then sent to the JGI for sequencing and assembly, also as previously described (5). Briefly, shotgun libraries were constructed for each of the SAGs from re-amplified MDA products and sequenced on an Illumina HiSeq2000. All general aspects of and detailed protocols for library construction and sequencing can be found on the JGI website (http://www.jgi.doe.gov/).

For assembly, raw sequence data was first passed through a filtering program developed at JGI to eliminate known sequencing and library preparation artifacts. Assembly was then performed using Velvet (6) and ALLPATHS-LG (7). Additional details of the assembly process have been previously described (5) and are available through the JGI Genome Portal (http://genome.jgi.doe.gov). Genome sequences are available through IMG (https://img.jgi.doe.gov/cgi-bin/mer/main.cgi). Genome-specific information can be accessed in both databases by searching for the IMG Taxon OIDs given in Table 1.

### Metagenome Sampling, Sequencing, Assembly, and Binning

Water samples were collected from two lakes, Lake Mendota (Madison, WI, USA) and Trout Bog (Vilas County, WI, USA). For Lake Mendota, depth-integrated water samples were collected from the top 12 meters at 96 time points during ice-free periods from 2008 to 2011. For Trout Bog, depth-integrated water samples were collected from the epilimnion (44 samples) and hypolimnion (45 samples) layers during ice-free periods from 2007 to 2009.

Sample collection, DNA sequencing, and metagenomic assembly, have been described previously (8, 9), as well as genomic binning for the Trout Bog samples (8). Similar genomic binning procedures were followed for the Lake Mendota samples. A summary is provided here.

All samples were filtered on 0.2 μm polyethersulfone filters (Supor, Pall Corp) prior to storage at -80°C, as described previously (8, 9). DNA was extracted from these filters using the FastDNA kit (MP Biomedicals) and sent to the JGI for sequencing, as described previously (8, 9).

Shotgun libraries were constructed for each of the samples and sequenced on an Illumina HiSeq2000, following a 2x150 indexed run recipe as previously described (8, 9). All general aspects of and detailed protocols for library construction and sequencing can be found on the JGI website (http://www.jgi.doe.gov/). Metagenomic sequence reads are publicly available on the JGI Genome Portal (http://genome.jgi.doe.gov/) under Proposal ID 394. The full list of metagenomes is given in Table S13.

Raw sequence data was passed through a filtering program developed at JGI to eliminate known sequencing and library preparation artifacts. Prior to assembly, reads were merged with FLASH (10), as previously described (8, 9). Merged reads were pooled by lake and layer into three co-assemblies using SOAPdenovo (11), and contigs from the resulting assemblies were assembled into a final assembly using Minimus (12), as previously described (8, 9). Additional details of the assembly process are available through the JGI Genome Portal (http://genome.jgi.doe.gov) under Proposal ID 394. The full list of assemblies is given in Table S14.

Genomes were binned from each metagenomic co-assembly using MetaBat (13), as described previously (8). Briefly, contigs were classified into bins using tetranucleotide frequency and coverage patterns across the time-series and then manually curated, as previously described (8). Genome sequences are available through IMG (https://img.jgi.doe.gov/cgi-bin/mer/main.cgi), and can be accessed by searching for the IMG Taxon OIDs given in Table 1.

Genomes were classified using taxonomic assignments from a set of 37 highly-conserved single-copy marker genes using Phylosift (14), as previously described (8). CheckM (15) was used to estimate genome completeness based on 204 single-copy marker genes conserved across the phylum Actinobacteria.

## Metatranscriptome Sampling and Sequencing

This study used four metatranscriptomes obtained as part of a 24-hour sampling experiment designed to identify diel trends in freshwater microbial communities. Samples were collected from the top of the water column (depth <1m) from Lake Mendota (Madison, WI, USA) on August 20 and 21, 2015. For each sample, between 200 and 400 mL lake water was filtered onto a 0.2 μm polyethersulfone filter (Supor, Pall Corp), flash frozen in liquid nitrogen, and stored at -80°C until extraction.

Samples were subject to TRIzol-based RNA extraction (Thermo Fisher Scientific, Waltham, MA) followed by phenol-chloroform separation and RNA precipitation. RNA was purified following an on-column DNAse digestion using the RNase-Free DNase Set (Qiagen, Venlo, Netherlands) and cleaned up with the RNeasy Mini Kit (Qiagen, Venlo, Netherlands). RNA was then sent to the University of Wisconsin-Madison Biotechnology Center (https://www.biotech.wisc.edu) for sequencing. There, samples were prepared for sequencing using the TruSeq RNA Library Prep Kit v2 (Illumina, San Diego, CA), with a ribosomal RNA (rRNA) depletion step using the Ribo-Zero rRNA Removal Kit (Bacteria) (Illumina). The resulting cDNA libraries were pooled in an equimolar ratio, and sequenced on an Illumina HiSeq2500 platform.

Raw paired-end reads were then trimmed using Sickle (16) and merged using FLASH (10). Sickle was run using default parameters, and FLASH was run with a maximum overlap of 100 nucleotides (M = 100). Finally, additional rRNA and ncRNA sequences were removed using SortMeRNA (17) using default parameters. SortMeRNA was run using eight built-in databases for bacterial, archaeal, and eukaryotic small and large ribosomal subunits and ncRNAs, derived from the SILVA 119 (18) and RFAM (19) databases.

Metadata about the four samples used in this study can be found in Table S1, and the raw RNA sequences can be found on the National Center for Biotechnology Information (NCBI) website under BioProject PRJNA362825. Additional information, including all protocols and scripts for sample collection, RNA extraction, sequencing, and bioinformatic analysis can be found on Github (https://github.com/McMahonLab/OMD-TOILv2, DOI:######).

## Genome Annotation, Metabolic Network Reconstruction, and Computation and Evaluation of Seed Compounds

Genome annotations were performed and metabolic network reconstructions were built using KBase. Contigs for each genome were uploaded to KBase and annotated using the “Annotate Microbial Contigs” method with default options, which uses components of the RAST toolkit for genome annotation (20, 21). Metabolic network reconstructions were obtained using the “Build Metabolic Model” app with default parameters, which relies on the Model SEED framework (22) to build a draft reconstruction.

Reconstructions were then pruned and converted to metabolic network graphs (Figure S3). During this process, exchange and transport reactions were removed from the reconstruction, to prevent extracellular metabolites from being identified as seed compounds. The biomass reaction was also removed, as KBase generates generalized biomass equations that may not reflect acI-specific biomass requirements. Finally, DNA/RNA replication reactions were removed, as these reactions do not represent metabolic processes. Reactions in the reconstructions were then mass- and charge-balanced. Next, currency metabolites (compounds used to carry electrons and functional groups) and highly-connected compounds (those that participate in many reactions, such as CO2 and O2) were removed to ensure paths in the resulting metabolic network graph would be biologically meaningful (23). Finally, the metabolic network graph was extracted from the reconstruction, to enable graph-theoretical identification of the network’s seed set.

Metabolic network reconstructions created from acI SAGs and MAGs will likely be missing reactions, as the underlying genomes are incomplete (Table 1). Previous studies have examined the effect of genome incompleteness on the predicted seed set (24). Using the formal (mathematical) definition of a seed compound, this showed that the percentage of correct seed compounds (true positives) is approximately equal to the completeness of the reaction network, and the number of false positives is approximately equal to the incompleteness of the network. Thus, we constructed composite genomes at higher taxonomic levels (e.g., tribe and clade) to increase genome completeness for more accurate seed identification at that taxonomic level (Figure S4). To do so, all genome-level metabolic network graphs for all genomes within each acI clade were combined to generate a composite clade-level metabolic network graph. Beginning with two genomes, nodes and edges unique to the second genome are identified and appended to the network graph for the first genome, giving a composite metabolic network graph. The process is repeated for each genome, until all of the network graphs have been incorporated into the composite.

Formally, the seed set of the network is defined as the minimal set of compounds that cannot be synthesized from other compounds in the network, and whose presence enables the synthesis of all other compounds in the network (24). Seed compounds for each composite clade-level metabolic network graph were calculated using the seed set framework (24) (Figure S5). Briefly, the graph is decomposed into its strongly connected components (SCCs), sets of nodes such that each node in the set is reachable from every other node. Seed compounds can then be found by identifying source components (components with no incoming edges) on the condensation of the original graph, a representation in which each SCC is represented as a single vertex. Here, each source component represents a seed set, and the nodes within that vertex represent seed compounds. If a seed set contains multiple seed compounds, each compound is assigned a weight equal to the size of the seed set. Because seed compounds are computed from a metabolic network, it is important to manually evaluate all predicted seed compounds to identify those that may be biologically meaningful, and do not arise from errors in the metabolic network reconstruction. Examples of this process are given below.

All steps were implemented using Python scripts, freely available as part of the reverseEcology Python package (https://pypi.python.org/pypi/reverseEcology/, DOI:######).

# Supplementary Results and Discussion

## Computation of Potential Seed Compounds

Metabolic network reconstructions for individual genomes contained between 110 and 339 genes, encoding between 241 and 587 reactions which interconvert between 374 and 699 metabolites (Table S15). On average, these genes account for 25% of the genes in the genome, a value consistent with metabolic network reconstructions for other organisms (25). Clade-level composite metabolic network graphs were larger, with between 602 and 811 metabolites (Table S16).

These composite metabolic network graphs contained a large number of disconnected components (groups of nodes that are not connected to the bulk of the network, Figure S6). For simplicity, these components were dropped from the graph, and seed compounds were computed for the single largest component. In all cases, the single largest component contained at least 80% of the nodes in the original graph (Table S16).

Decomposition of the three metabolic network graphs into their strongly connected components (SCCs) resulted in a bow-tie structure, in which a single giant component contains a substantial fraction of the compounds (Figure S6). Across the three clades, the giant component contained 61% of the metabolites, a larger fraction than reported for other organisms (26), which may be a consequence of the small and streamlined genomes of acI bacteria.

The total number of predicted seed sets (source components in the SCC condensation) ranged from 63 to 95, and the number of seed compounds ranged from 70 to 102 (Table S16). This discrepancy arises because some seed sets contain multiple compounds (Table S17). However, such seed sets were rare (4% of all seed sets), and contained at most six compounds (Table S17). The majority of seed compounds (96%) belonged to seed sets containing only a single compound (Table S17).

## Evaluation of Potential Seed Compounds

Here, we present a series of brief vignettes explaining why particular compounds were retained or discarded based on their biological (im)plausibility. For biologically plausible compounds, we also provide examples of manual curation efforts. These vignettes are not intended to provide a comprehensive explanation for all compounds, but to provide a flavor of the types of decisions that went into accepting or rejecting particular compounds.

**Carbamoyl phosphate**. Carbamoyl phosphate was predicted as a seed compound for all three clades. Carbamoyl phosphate synthase is the first step in arginine and pyrimidine biosynthesis, and catalyzes the reaction:

2 ATP + L-glutamine + hydrogen carbonate + H2O → L-glutamate + carbamoyl phosphate + 2 ADP + phosphate + 2 H+

This reaction contains a number of currency metabolites (ATP, ADP glutamine, glutamate), as well as the highly-connected metabolites carbonate, water, phosphate and protons. All of these metabolites were removed from the network during pruning. Thus, the reaction responsible for carbamoyl phosphate synthesis was effectively removed from the network, rendering carbamoyl phosphate a seed compound. Manual inspection of individual genomes revealed the gene for carbamoyl phosphate synthase, confirming carbamoyl phosphate is not an auxotrophy.

**R-enoyl-ACP**. A number of R-enoyl-ACP compounds were predicted to be seed compounds in clades acI-A and acI-B. These compounds were associated with a single COG annotated as an “Enoyl-[acyl-carrier-protein] reductase,” the enzyme which catalyzes the final step in fatty acid elongation. Many other seed compounds were predicted to participate in fatty acid and phospholipid biosynthesis, including a number of saturated fatty acids (associated with a COG annotated as a “long-chain-fatty-acid–CoA ligase”) and 1-acyl-sn-glycerol 3-phosphate compounds (associated with a COG annotated as an “1-acyl-sn-glycerol-3-phosphate acyltransferase”). Given the broad substrate specificity of these enzymes, KBase automatically assigns these enzymes to the catalysis of a number of reactions. Fatty acid and phospholipid biosynthesis pathways are often organism-specific and unlikely to be properly annotated by automatic metabolic reconstruction pipelines. Thus, these compounds were excluded from further consideration.

**L-Aspartate-4-semialdehyde, L-homoserine, and O-Phospho-L-homoserine.** Clade acI-C was predicted to have a seed set containing these three compounds. These three compounds can be interconverted via the following reactions:

homoserine dehydrogenase: L-aspartate-4-semialdehyde <–> L-homoserine

homoserine kinase: L-homoserine <–> O-phospho-L-homoserine

making the three compounds members of a SCC. The first reaction, homoserine dehydrogenase, is the final step in homoserine biosynthesis, so these compounds suggest an auxotrophy for homoserine. Homoserine biosynthesis proceeds via the following reactions:

aspartate kinase: aspartate –> L-aspartyl-4-phosphate

aspartate semialdehyde dehydrogenase: L-aspartyl-4-phosphate –> L-aspartate-4-semialdehyde

homoserine dehydrogenase: L-aspartate-4-semialdehyde –> homoserine

The presence of L-aspartate-4-semialdehyde as a seed compound suggests the reaction “aspartate semialdehyde dehydrogenase” is missing, and were unable to manually identify a candidate gene for this reaction. Furthermore, the acI-C composite genome contains the other two reactions in the pathway. Thus, on the evidence available, we conclude acI-C is auxotrophic for homoserine.

**L-arogenate.** This compound was predicted as a seed compound for clade acI-C, suggesting an auxotrophy for tyrosine. Tyrosine can be synthesized via the following reactions:

chorismate mutase: chorismate –> prephenate

prephenate aminotransferase: prephanate –> L-arogenate

arogenate dehydrogenase: L-arogenate –> L-tyrosine

L-arogenate was predicted to be a seed compound based on the presence of “arogenate dehydrogenase”, the final step in the pathway. The reaction “chorismate mutase” is also present, but we were unable to find a candidate gene for the reaction “prephenate aminotransferase,” suggesting an auxotrophy for tyrosine. However, L-tyrosine can be synthesized from chorismate via an alternative pathway:

chorismate mutase: chorismate –> prephenate

prephenate dehydrogenase: prephenate –> 4-hydroxyphenylpyruvate

tyrosine aminotransferase: 4-hydroxyphenylpyruvate –> L-tyrosine

All three genes in this pathway are present in the genome, indicating acI-C is not auxotrophic for tyrosine.

**Ala-Leu and gly-pro-L**. These di-peptides were predicted to be seed compounds for all three clades. The compounds are associated with the following reactions:

H2O + Ala-Leu –> L-Leucine + L-Alanine

H2O + Gly-Pro –> Glycine + L-Proline

These reactions were associated with four COGs, annotated as aminopeptidases. These seed compounds suggest the ability for the acI to degrade peptides, but the broad specificity of aminopeptidases means these particular di-peptides are unlikely to be the only substrates. Similarly, a number of seed compounds were associated with COGs annotated as gluco- and galactosidases, which also have broad substrate specificity. As a result, these genes were further investigated as described in the primary manuscript.

# References

1. Martinez-Garcia M, Swan BK, Poulton NJ, Gomez ML, Masland D, Sieracki ME, Stepanauskas R. 2012. High-throughput single-cell sequencing identifies photoheterotrophs and chemoautotrophs in freshwater bacterioplankton. The ISME Journal 6:113–123.

2. Garcia SL, McMahon KD, Martinez-Garcia M, Srivastava A, Sczyrba A, Stepanauskas R, Grossart H-P, Woyke T, Warnecke F. 2013. Metabolic potential of a single cell belonging to one of the most abundant lineages in freshwater bacterioplankton. The ISME Journal 7:137–147.

3. Newton RJ, Jones SE, Eiler A, McMahon KD, Bertilsson S. 2011. A guide to the natural history of freshwater lake bacteria. Microbiology and Molecular Biology Reviews 75:14–49.

4. Ludwig W, Strunk O, Westram R, Richter L, Meier H, Yadhukumar, Buchner A, Lai T, Steppi S, Jobb G, Förster W, Brettske I, Gerber S, Ginhart AW, Gross O, Grumann S, Hermann S, Jost R, König A, Liss T, Lüssmann R, May M, Nonhoff B, Reichel B, Strehlow R, Stamatakis A, Stuckmann N, Vilbig A, Lenke M, Ludwig T, Bode A, Schleifer K-H. 2004. ARB: a software environment for sequence data. Nucleic Acids Research 32:1363–1371.

5. Ghylin TW, Garcia SL, Moya F, Oyserman BO, Schwientek P, Forest KT, Mutschler J, Dwulit-Smith J, Chan L-K, Martinez-Garcia M, Sczyrba A, Stepanauskas R, Grossart H-P, Woyke T, Warnecke F, Malmstrom RR, Bertilsson S, McMahon KD. 2014. Comparative single-cell genomics reveals potential ecological niches for the freshwater acI Actinobacteria lineage. The ISME Journal 8:2503–2516.

6. Zerbino DR, Birney E. 2008. Velvet: Algorithms for de novo short read assembly using de Bruijn graphs. Genome Research 18:821–829.

7. Gnerre S, Maccallum I, Przybylski D, Ribeiro FJ, Burton JN, Walker BJ, Sharpe T, Hall G, Shea TP, Sykes S, Berlin AM, Aird D, Costello M, Daza R, Williams L, Nicol R, Gnirke A, Nusbaum C, Lander ES, Jaffe DB. 2011. High-quality draft assemblies of mammalian genomes from massively parallel sequence data. Proceedings of the National Academy of Sciences 108:1513–1518.

8. Bendall ML, Stevens SLR, Chan L-K, Malfatti S, Schwientek P, Tremblay J, Schackwitz W, Martin J, Pati A, Bushnell B, Froula J, Kang D, Tringe SG, Bertilsson S, Moran MA, Shade AL, Newton RJ, McMahon KD, Malmstrom RR. 2016. Genome-wide selective sweeps and gene-specific sweeps in natural bacterial populations. The ISME Journal 10:1589–1601.

9. Garcia SL, Buck M, McMahon KD, Grossart H-P, Eiler A, Warnecke F. 2015. Auxotrophy and intra-population complementary in the ‘interactome’ of a cultivated freshwater model community. Molecular Ecology 24:4449–4459.

10. Magoc T, Salzberg SL. 2011. FLASH: fast length adjustment of short reads to improve genome assemblies. Bioinformatics 27:2957–2963.

11. Luo R, Liu B, Xie Y, Li Z, Huang W, Yuan J, He G, Chen Y, Pan Q, Liu Y, Tang J, Wu G, Zhang H, Shi Y, Liu Y, Yu C, Wang B, Lu Y, Han C, Cheung DW, Yiu S-M, Peng S, Xiaoqian Z, Liu G, Liao X, Li Y, Yang H, Wang J, Lam T-W, Wang J. 2012. SOAPdenovo2: an empirically improved memory-efficient short-read de novo assembler. GigaScience 1:18.

12. Sommer DD, Delcher AL, Salzberg SL, Pop M. 2007. Minimus: a fast, lightweight genome assembler. BMC Bioinformatics 8:64.

13. Kang DD, Froula J, Egan R, Wang Z. 2015. MetaBAT, an efficient tool for accurately reconstructing single genomes from complex microbial communities. PeerJ 3:e1165.

14. Darling AE, Jospin G, Lowe E, Matsen FA, Bik HM, Eisen JA. 2014. PhyloSift: phylogenetic analysis of genomes and metagenomes. PeerJ 2:e243.

15. Parks DH, Imelfort M, Skennerton CT, Hugenholtz P, Tyson GW. 2015. CheckM: assessing the quality of microbial genomes recovered from isolates, single cells, and metagenomes. Genome Research 25:1043–1055.

16. Joshi NA, Fass JN. 2011. Sickle: A sliding-window, adaptive, quality-based trimming tool for FastQ files (Version 1.33).

17. Kopylova E, Noe L, Touzet H. 2012. SortMeRNA: fast and accurate filtering of ribosomal RNAs in metatranscriptomic data. Bioinformatics 28:3211–3217.

18. Quast C, Pruesse E, Yilmaz P, Gerken J, Schweer T, Yarza P, Peplies J, Glöckner FO. 2013. The SILVA ribosomal RNA gene database project: improved data processing and web-based tools. Nucleic Acids Research 41:D590–D596.

19. Nawrocki EP, Burge SW, Bateman A, Daub J, Eberhardt RY, Eddy SR, Floden EW, Gardner PP, Jones TA, Tate J, Finn RD. 2015. Rfam 12.0: Updates to the RNA families database. Nucleic Acids Research 43:D130–D137.

20. Brettin T, Davis JJ, Disz T, Edwards RA, Gerdes S, Olsen GJ, Olson R, Overbeek RA, Parrello B, Pusch GD, Shukla M, Thomason JA, Stevens R, Vonstein V, Wattam AR, Xia F. 2015. RASTtk: a modular and extensible implementation of the RAST algorithm for building custom annotation pipelines and annotating batches of genomes. Scientific Reports 5:8365.

21. Overbeek RA, Olson R, Pusch GD, Olsen GJ, Davis JJ, Disz T, Edwards RA, Gerdes SY, Parrello B, Shukla M, Vonstein V, Wattam AR, Xia F, Stevens RL. 2014. The SEED and the Rapid Annotation of microbial genomes using Subsystems Technology (RAST). Nucleic Acids Research 42:206–214.

22. Henry CS, DeJongh M, Best AA, Frybarger PM, Linsay B, Stevens RL. 2010. High-throughput generation, optimization and analysis of genome-scale metabolic models. Nature Biotechnology 28:977–982.

23. Ma H, Zeng A-P. 2003. Reconstruction of metabolic networks from genome data and analysis of their global structure for various organisms. Bioinformatics 19:270–277.

24. Borenstein E, Kupiec M, Feldman MW, Ruppin E. 2008. Large-scale reconstruction and phylogenetic analysis of metabolic environments. Proceedings of the National Academy of Sciences 105:14482–14487.

25. Feist AM, Herrgård MJ, Thiele I, Reed JL, Palsson BØ. 2009. Reconstruction of biochemical networks in microorganisms. Nature Reviews Microbiology 7:129–143.

26. Ma HW, Zeng A-PP. 2003. The connectivity structure, giant strong component and centrality of metabolic networks. Bioinformatics 19:1423–1430.
